# The Analogs of Furanyl Methylidene Rhodanine Exhibit Broad-Spectrum Inhibitory and Inactivating Activities against Enveloped Viruses, including SARS-CoV-2 and Its Variants

**DOI:** 10.3390/v14030489

**Published:** 2022-02-27

**Authors:** Jing Pu, Xiaoyang He, Wei Xu, Cong Wang, Qiaoshuai Lan, Chen Hua, Kai Wang, Lu Lu, Shibo Jiang

**Affiliations:** 1 Key Laboratory of Medical Molecular Virology (MOE/NHC/CAMS), School of Basic Medical Sciences, Shanghai Institute of Infectious Disease and Biosecurity, Biosafety Level 3 Laboratory, Shanghai Medical College, Shanghai Frontiers Science Center of Pathogenic Microbes and Infection, Fudan University, Shanghai 200438, China; 17111010015@fudan.edu.cn (J.P.); xuwei11@fudan.edu.cn (W.X.); congwang1111@163.com (C.W.); 18111010010@fudan.edu.cn (Q.L.); 16211010047@fudan.edu.cn (C.H.); 16111010090@fudan.edu.cn (K.W.); 2 Beijing Institute of Radiation Medicine, Beijing 200032, China; hexiaoyang@aliyun.com

**Keywords:** small-molecule compound, enveloped virus, inhibitor, inactivator, SARS-CoV-2

## Abstract

In recent years, infectious diseases caused by viral infections have seriously endangered human health, especially COVID-19, caused by SARS-CoV-2, which continues to spread worldwide. The development of broad-spectrum antiviral inhibitors is urgently needed. Here, we report a series of small-molecule compounds that proved effective against human coronaviruses (HCoV), such as SARS-CoV-2 and its variants of concern (VOCs), including Alpha (B.1.1.7), Beta (B.1.351), Gamma (P.1), Delta (B.1.617.2), and Omicron (B.1.1.529), SARS-CoV, MERS-CoV, HCoV-OC43, and other viruses with class I viral fusion proteins, such as influenza virus, Ebola virus (EBOV), Nipah virus (NiV), and Lassa fever virus (LASV). They are also effective against class II enveloped viruses represented by ZIKV and class III enveloped viruses represented by vesicular stomatitis virus (VSV). Further studies have shown that these compounds may exert antiviral effects through a variety of mechanisms, including inhibiting the formation of the six-helix bundle, which is a typical feature of enveloped virus fusion with cell membranes, and/or targeting viral membrane to inactivate cell-free virions. These compounds are expected to become drug candidates against SARS-CoV-2 and other enveloped viruses.

## 1. Introduction

Since the beginning of this century, infectious diseases caused by enveloped viruses, including severe acute respiratory syndrome CoV (SARS-CoV), Middle East respiratory syndrome coronavirus (MERS-CoV), and SARS-CoV-2, as well as other enveloped viruses, such as influenza virus H1N1, Ebola virus (EBOV), and Zika virus (ZIKV), have severely affected human life and health [1,2,3,4,5]. In particular, COVID-19, caused by SARS-CoV-2, is spreading rapidly around the world, and its impact on humans is unprecedented. This pandemic has caused more than 373 million infections and 5.68 million deaths worldwide as of 30 January 2022 (https://www.who.int/emergencies/diseases/novel-coronavirus-2019) (accessed on 30 January 2022). Some prophylactic vaccines have been approved to slow down this pandemic [6,7,8,9,10,11,12,13]. For COVID-19 treatment, the small-molecule compounds remdesivir [14] and molnupiravir [15] have been approved for clinical use by the US Food and Drug Administration (FDA) and the UK Medicines and Healthcare products Regulatory Agency (MHRA), respectively. However, the continuous emergence of SARS-CoV-2 variants and infectious diseases caused by influenza virus, ZIKV, and HIV calls for the urgent development of inhibitors with broad-spectrum activity.

We previously reported the compound 11d (furanyl methylidene rhodanine) with potent anti-HIV-1 activity [16]. Here, we renamed it FD001 and found that it could effectively inhibit the infection of enveloped viruses, including SARS-CoV-2 and its variants, but not the infection of nonenveloped viruses, such as human papilloma virus (HPV) and human enterovirus 71 virus (EV71). At the same time, we designed a series of novel furanyl methylidene rhodanine analogs, some of which had a higher selection index (SI) toward different viruses. This series of compounds not only inhibits viral infection of cells, but also inactivates cell-free virions. Further mechanism studies showed that they target the viral membrane and are able to block the formation of the six-helix bundle (6-HB) fusion core of some enveloped viruses, such as HIV-1. This multi-mechanism action may lead to the excellent antiviral effect of this series of compounds. Therefore, the development of broad-spectrum anti-enveloped virus inhibitors is expected not only to deal with SARS-CoV-2, but also to provide a drug reserve for other enveloped viruses that may break out in the future.

## 2. Materials and Methods

### 2.1. Synthesis and Characterization of Novel Furanyl Methylidene Rhodanine Analogs FD007–010 and FD012–013

**Scheme 1:**
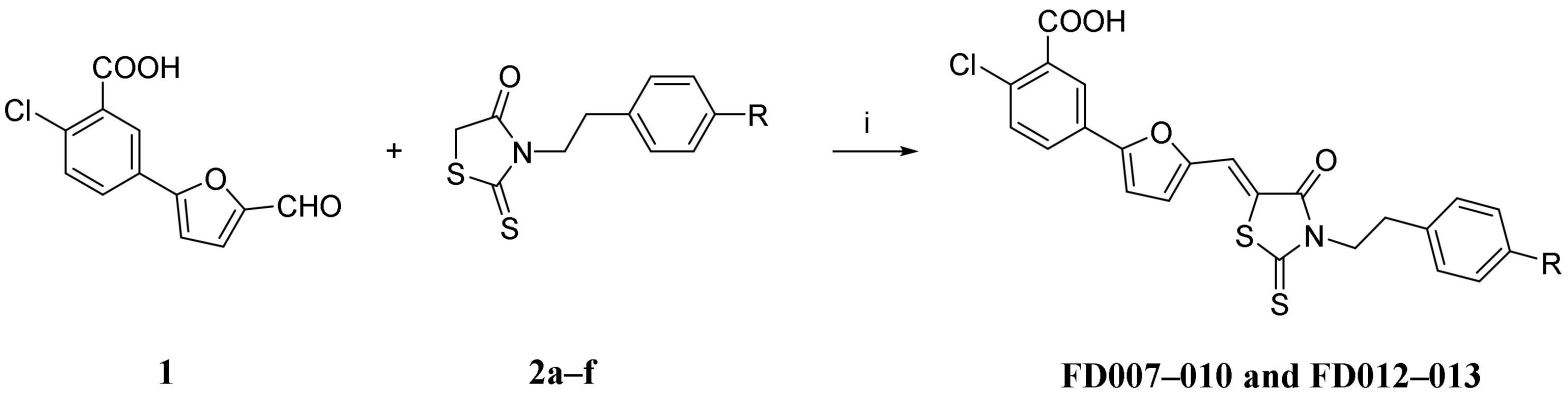
The synthesis procedure for FD001 analogs FD007–010 and FD012–013. Reagents and conditions: (i) CH_3_CO_2_NH_4_, EtOH, reflux, 2 h.

**Scheme 2:**
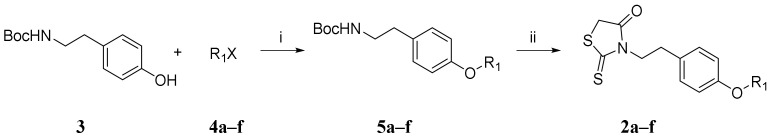
The synthesis procedure of rhodanine building blocks 2a–f. Reagents and conditions: (i) K_2_CO_3_, DMF, 80 °C, 12 h; (ii) (1) AcCl, MeOH, r.t., 24 h; (2) bis(carboxymethyl) trithiocarbonate, Et3N, i-PrOH, MW, 90 °C, 45 min.

The procedure to synthesize the novel furanyl methylidene rhodanine analogs FD007–010 and FD012–013 (Table 1) was performed by Knoevenagel condensation of aldehyde 1 with *N*-substituted rhodanines 2a–f which contained various alkyl or aryl motifs at the terminus of phenyl (Scheme 1). Firstly, as shown in Scheme 2, alkylation of *N*-Boc-tyramine 3 with corresponding bromides (4a, 4c–f) or mesylate 4b gave *N*-Boc-tyramine derivatives 5a–f in moderate yield. Subsequently, AcCl–MeOH-mediated deprotection of the Boc group of 5a–f was followed by rhodanine formation with bis(carboxymethyl) trisulfide carbonate under microwave at 90 °C for 45 min to afford the rhodanine building blocks 2a–f in satisfactory yield over two steps. Then, Knoevenagel condensation of 2a–b with aldehyde 1 in EtOH in the presence of a catalytic amount of ammonium acetate at reflux temperature for 2 h was done to conveniently afford the requisite FD001 analogs in acceptable yield. More detailed information is provided in the Supplemental Materials.

### 2.2. Cells and Virus

The 293T, Huh-7, Caco2, Vero E6, MDCK, HeLa, and RD cells were all obtained from the American Type Culture Collection (ATCC). U87 and TZM-bl cells were obtained from the NIH AIDS Reagent Program. Cells were cultured in Dulbecco’s modified Eagle’s medium (DMEM) with 10% fetal bovine serum (FBS), together with penicillin and streptomycin, and maintained at 37 °C under a 5% CO_2_ incubator. The authentic SARS-CoV-2 wildtype (WT) nCoV-SH01 and Delta variant were isolated from COVID-19 patients by researchers working in a BSL-3 Laboratory of Fudan University.

### 2.3. Inhibition of Pseudovirus (PsV) Infection

Inhibitory activity against PsV infection was measured according to the previously reported method [17,18,19]. Briefly, 50 μL of serially diluted compounds or PBS was incubated with 50 μL of PsV (100 TCID50) for 30 min at 37 °C, and the corresponding target cells (hACE2-293T cells for PsV of SARS-CoV, SARS-CoV-2; Caco2 cells for PsV of SARS-CoV-2 variants; Huh-7 cells for PsV of MERS-CoV, vesicular stomatitis virus (VSV), EBOV, and Lassa fever virus (LASV); MDCK cells for H5N1 and H7N9 PsV; HeLa cells for PsV of human papilloma virus (HPV); U87 cells for PsV of Nipah virus (NiV) (2 × 10^5^/mL, 100 μL)) were added to the mixture. After incubating in a 37 °C, 5% CO_2_ incubator for 48 h, supernatants were discarded, and cells were washed twice with PBS. Luciferase activity was measured according to the instructions of the luciferase assay reagent (Promega, Madison, WI, USA), and the Infinite M200PRO microplate reader (Tecan, NC, USA) was used as a luminescence counter.

### 2.4. Cytotoxicity

Cytotoxicity of the compounds on different cells was tested as described in the Cell Counting Kit-8 (CCK-8; Dojindo, Kumamoto, Kyushu, Japan) instructions. Briefly, 50 μL of serially diluted compounds were added to 100 µL of target cells (2 × 10^5^/mL). After incubation in a 37 °C, 5% CO_2_ incubator for 48 h, supernatants were discarded, and 100 µL of fresh medium and 5 µL of CCK-8 were added. After an additional 4 h incubation at 37 °C, the absorbance at 450 nm was measured using the Infinite M200PRO microplate reader (Tecan, NC, USA).

### 2.5. Inhibition of Authentic Virus Infection

The plaque reduction assay was used to test the inhibitory activity of compounds against infection by authentic SARS-CoV-2 WT (nCoV-SH01), Delta variant, and ZIKV in Vero-E6 cells, as well as influenza virus H3N2 and H1N1 in MDCK cells, as previously described [20,21,22]. Briefly, ZIKV PRVABC59 (2015/Puerto Rico), H3N2 (A/Victoria/361/2011), or H1N1 (A/California/04/2009) with 0.001 MOI, SARS-CoV-2 WT (nCoV-SH01) with 0.01 MOI, or Delta variant with 0.0025 MOI was incubated with serially diluted compounds or PBS control at 37 °C for 1 h (for influenza virus, 2 µg/mL TPCK-trypsin was required). Then, the mixture was added to a 24-well plate or a 96-well plate seeded with target cells and incubated at 37 °C for 2 h. The cells were washed twice with PBS and covered with DMEM containing 1% low-melting agarose (if influenza virus, 2 µg/mL TPCK-trypsin was required) or DMEM containing 1% carboxymethyl cellulose and 2% FBS. This was followed by incubation at 37 °C for 3 to 5 days and then staining with 1% crystal violet.

The cytopathic effect (CPE) reduction assay was used to detect the ability of compounds to inhibit human enterovirus 71 virus (EV71) or HCoV-OC43 infection of RD cells, as previously described [22]. Briefly, the virus was incubated with serially diluted compounds or PBS control at 37 °C for 30 min. Then, the mixture was added to target cells (2 × 10^5^/mL, 100 µL). Culture at 37 °C continued until obvious cytopathic effects were observed in the PBS control group. The inhibitory activity of compounds was measured using CCK-8 as described above. Information on cell lines, virus prototype strains, virus inoculation TCID_50_, and culture conditions is provided in Supplementary Table S1.

### 2.6. Inactivation of Cell-Free Virions

The ability of compounds to inactivate cell-free virions was measured as previously described [23]. Briefly, different concentrations of compound or PBS were incubated with 400 TCID_50_ PsV at 4 °C for 1 h. Then, PEG-6000 was added to the mixture with a final concentration of 3% and incubated at 4 °C for another 1 h. After centrifuging the mixture at 13,000 rpm at 4 °C for 30 min, the supernatant was discarded, and the pellet was washed twice by 3% PEG-6000 containing 10 mg/mL BSA. Finally, the pellet was resuspended in 100 µL of medium and added to 100 µL of 2 × 10^5^/mL target cells. After incubating at 37 °C for 2 days, luciferase activity was measured.

### 2.7. Inhibition of HIV-1 6-HB Formation

ELISA was used to measure the inhibitory activity of HIV-1 6-HB formation as previously described [24]. Briefly, 50 μL of HIV-1 6-HB-specific 2G8 mAb at 4 μg/mL in 0.1 M sodium bicarbonate buffer (pH 8.6) was added to the ELISA plate and incubated at 4 °C overnight. The plate was washed with PBS containing 0.1% Tween-20 (PBS-T) and then blocked with PBS containing 5% BSA, followed by incubation at 37 °C for 2 h and three washes. The serially diluted compounds were incubated with N63 peptide (50 μL, final concentration 1 μM) at 37 °C for 30 min, and then C34-bio (100 μL, final concentration 1 μM) was added, followed by incubation at 37 °C for another 30 min. Next, the mixture (50 μL/well) was transferred to the 2G8-coated ELISA plate and incubated at 37 °C for 1 h. SA-HRP and TMB were added successively. Then, the optical density at 450 nm (OD_450_) was recorded using a Tecan microplate reader (Maännedorf, Switzerland).

The secondary structure of the FD-compound in complex with N46 peptide or C34 peptide was measured by circular dichroism (CD) spectroscopy, as previously described [25]. In brief, the FD-compound, N46 peptide, and C34 peptide were all dissolved in ddH_2_O. Then, the FD-compound was first incubated with N46 peptide at 37 °C for 30 min, followed by the addition of C34 peptide and incubation at 37 °C for another 30 min. Final concentrations of the N46 peptide, C34 peptide, and FD-compound were 10 μM and 200 μM, respectively. The mixture was then tested on a Jasco spectropolarimeter (Model J-815; Jasco, Inc., Easton, MD, USA) using a 1-nm bandwidth with a 1 nm step resolution from 195 to 260 nm at 25 °C. The α-helical content was obtained as follows: (mean residue ellipticity (θ) at 222 nm)/(−33,000 degrees cm^2^·dmol^−1^) × 100%.

### 2.8. Sucrose Density Gradient Assay to Evaluate Viral Inactivation

This experiment was performed as previously described [26]. In brief, HIV-1 Bal.01 PsV was treated with PBS containing 1% (*v*/*v*) DMSO, 50 μM FD001, 50 μM FD-9170 peptide, or PBS containing 1% (*v*/*v*) Triton X-100 at 37 °C for 2 h. Then, the treated virions were gently loaded to the top of ultracentrifuge tubes containing different concentrations of sucrose solutions (from bottom to top, 70%, 60%, 50%, 40%, 30%, and 20%) and then centrifuged in a swinging bucket rotor (SW41Ti, Beckman Coulter, Brea, CA, USA) in an Optima L-100 XP ultracentrifuge (Beckman Coulter) at 30,000 rpm at 4 °C for 2.5 h. Fractions from top to bottom were collected and detected for viral p24 antigen with 183 mAb by Western blot.

## 3. Results

### 3.1. Inhibitory and Inactivating Activity of FD001 and Analogs on Infection of SARS-CoV-2 and Its Variants

**Figure 1:**
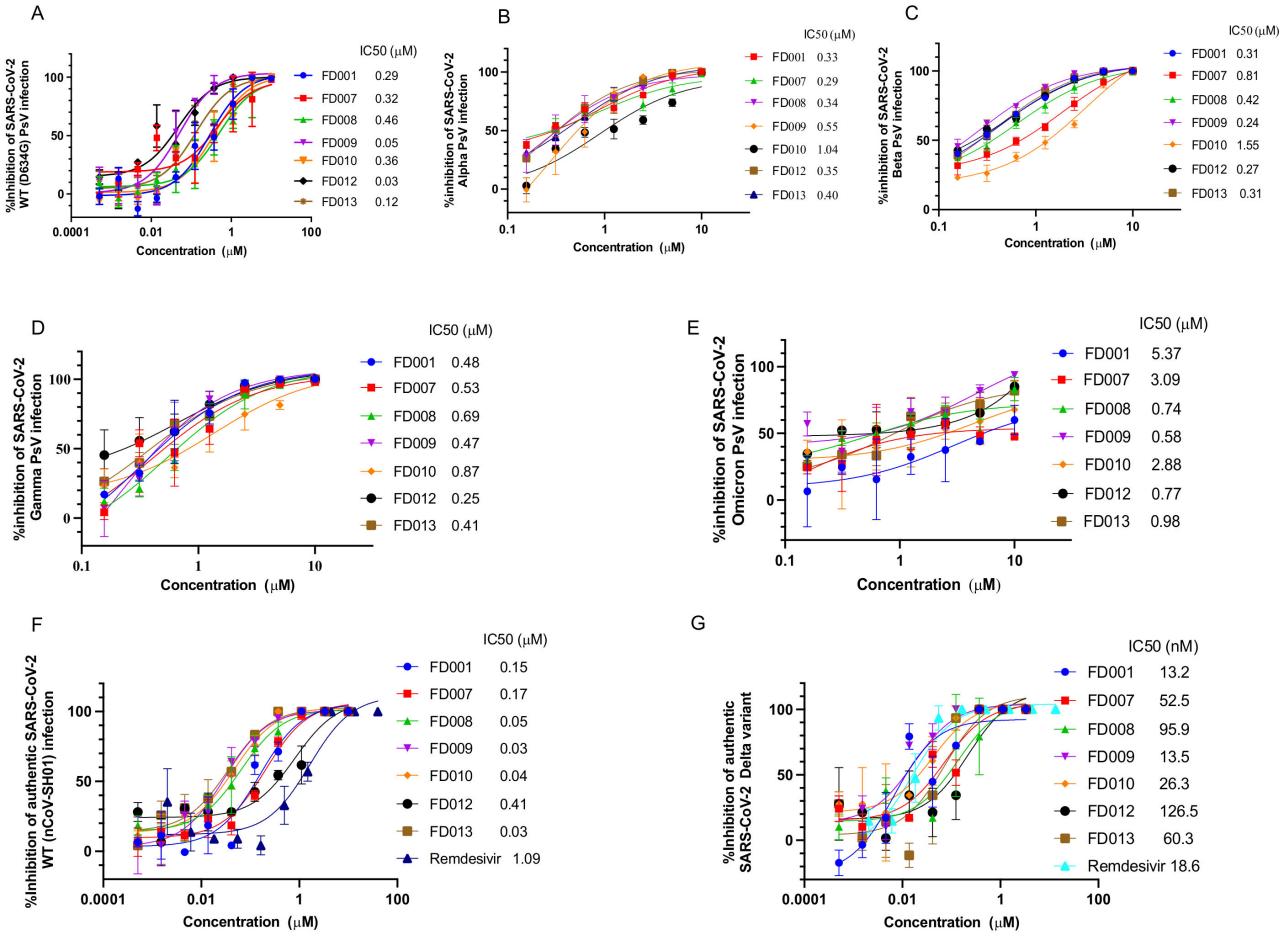
FD-compounds effectively inhibited infection of SARS-CoV-2 and its variants. (**A**) FD-compounds inhibited SARS-CoV-2 PsV infection in hACE2-293T cells; (**B**–**E**) FD-compounds inhibited infection of pseudotyped SARS-CoV-2 Alpha (B.1.1.7), Beta (B.1.351), Gamma (P.1), and Omicron (B.1.1.529) in Caco2 cells; (**F**,**G**) the plaque reduction assay was used to detect the inhibitory activity of FD-compounds against infection of authentic SARS-CoV-2 WT (MOI = 0.01) and Delta (B.1.617.2) variant in Vero E6 cells (MOI = 0.0025). Data are means ± SD of triplicate samples from a representative experiment.

Previously, we found that FD001 acted as a fusion inhibitor against HIV-1 [16]. Here, we were surprised to find that FD001 and its analogs effectively inhibited SARS-CoV-2 PsV from infecting hACE2-293T cells (Figure 1A). Furthermore, we found that all of these compounds were effective in inhibiting infection caused by SARS-CoV-2 variants in a concentration-dependent manner, including B.1.1.7 (Alpha), B.1.351 (Beta), P.1 (Gamma), and B.1.1.529 (Omicron) PsV (Figure 1B–E). In addition, similarly to remdesivir, FD001 and its analogs proved effective against infection of Vero E6 cells by authentic SARS-CoV-2 WT (nCoV-SH01) (MOI = 0.01) and Delta variant (MOI = 0.0025) with IC_50_ values at the nM level (Figure 1F,G).

**Figure 2:**
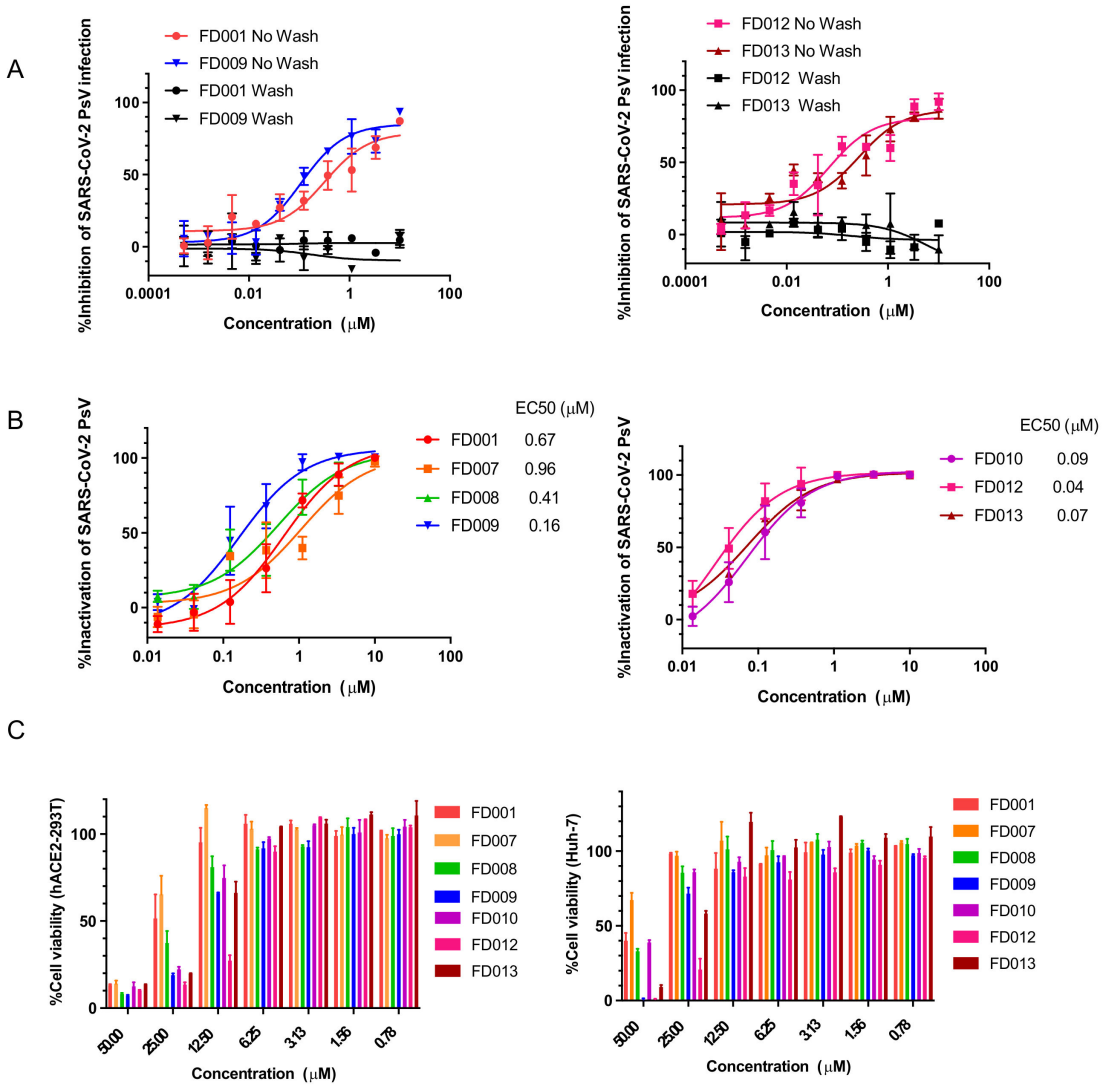
FD-compounds exerted antiviral activity by targeting cell-free virions, rather than the cells. (**A**) FD-compounds exhibited inhibitory activity against infection of SARS-CoV-2 PsV when they were preincubated with hACE2-293T cells at 4 °C without washes, but no inhibitory activity after the compounds were washed out, before addition of PsV; (**B**) FD-compounds inactivated cell-free SARS-CoV-2 PsV; (**C**) cytotoxicity of FD-compounds on hACE2-293T and Huh-7 cells. Data are means ± SD of triplicate samples from a representative experiment.

Inhibition of PsV infection indicates that the compound acts at the stage before viral entry to target cells and that it may act on target cells, cell-free virions, or the cell–virus interaction process to exert antiviral activity. First, we tested whether these compounds could interact with host cells. Compounds with higher SI values, such as FD001, FD009, FD012, and FD013, were selected for the experiment. To achieve this, target cells were first incubated with compounds at 4 °C for 30 min. Then, the unbound compounds were washed away, virus was added, and the unwashed group was used as a control. Results showed that these compounds were not effective in inhibiting viral infection after washing, indicating that the compounds did not bind to target cells to exert antiviral activity (Figure 2A). To further investigate whether the compounds act on cell-free virions, we incubated them with SARS-CoV-2 PsV at 4 °C for 1 h and separated compounds from virus using PEG-6000. After this, the infectivity of the virus was measured. Results showed that the cell-free virions lost their infectivity after compound pretreatment (Figure 2B). These results indicate that the compounds may exert antiviral activity by targeting virions. In order to more fully evaluate the characteristics of these compounds, we tested their cytotoxicity on different cells. Almost all compounds, except FD012, had no significant cytotoxicity on hACE2-293T and Huh-7 cells at a concentration of 12.5 µM (Figure 2C). Therefore, the above results indicate that FD-compounds inactivate cell-free virions and have no obvious cytotoxicity within the active concentration range.

### 3.2. Inhibitory and Inactivating Activity of FD-Compounds on SARS-CoV and MERS-CoV Infection

In the past 20 years, the emergence of human highly pathogenic coronaviruses SARS-CoV [27,28] and MERS-CoV [29] has caused widespread transmission. Human SARS-CoV has 82% nucleotide identity with SARS-CoV-2 [30]. We then tested the antiviral activity of FD-compounds against SARS-CoV PsV. Monoclonal antibody 33G4, which targets the RBD region of SARS-CoV and blocks the binding of the receptor [31], served as a positive control. As shown in Table 2, most of these FD-compounds inhibited SARS-CoV PsV infection and inactivated cell-free SARS-CoV PsV at the nanomolar level. In particular, the inhibitory activity of FD012 was nearly 10-fold higher than that of FD001 with an IC_50_ as low as 0.02 µM. The SI values of FD009 and FD012 against SARS-CoV PsV were up to 579 and 615, respectively (Table 2). Similarly, we also evaluated the inhibitory and inactivating activities of these compounds against MERS-CoV PsV. As shown in Table 2, similar to results on SARS-CoV PsV and SARS-CoV-2 PsV, all FD-compounds effectively inhibited and inactivated MERS-CoV PsV with SI values of FD010 and FD013 up to 1117 and 1030, respectively.

FD001 and FD012 could also effectively inhibit infection by HCoV-OC43 with IC_50_ values of 0.48 and 0.11 μM, respectively (Supplementary Figure S1).

### 3.3. FD-Compounds Effectively Inhibited Infection by Other Enveloped Viruses with Class I Viral Fusion Proteins

Further study demonstrated that these compounds effectively inhibited influenza virus, including H5N1 and H7N9 PsV (Supplementary Table S2), as well as authentic H3N2 and H1N1 infection (Supplementary Figure S2). FD001 and FD012 were further tested for their inhibitory activity against other viruses with class I viral fusion proteins. The results show that they could effectively inhibit infection by the pseudotyped EBOV, NiV, and LASV (Supplementary Table S3).

### 3.4. FD-Compounds Effectively Inhibited Infection by Enveloped Viruses with Class II and III Viral Fusion Protein, but Not Nonenveloped Virus Infection

**Figure 3:**
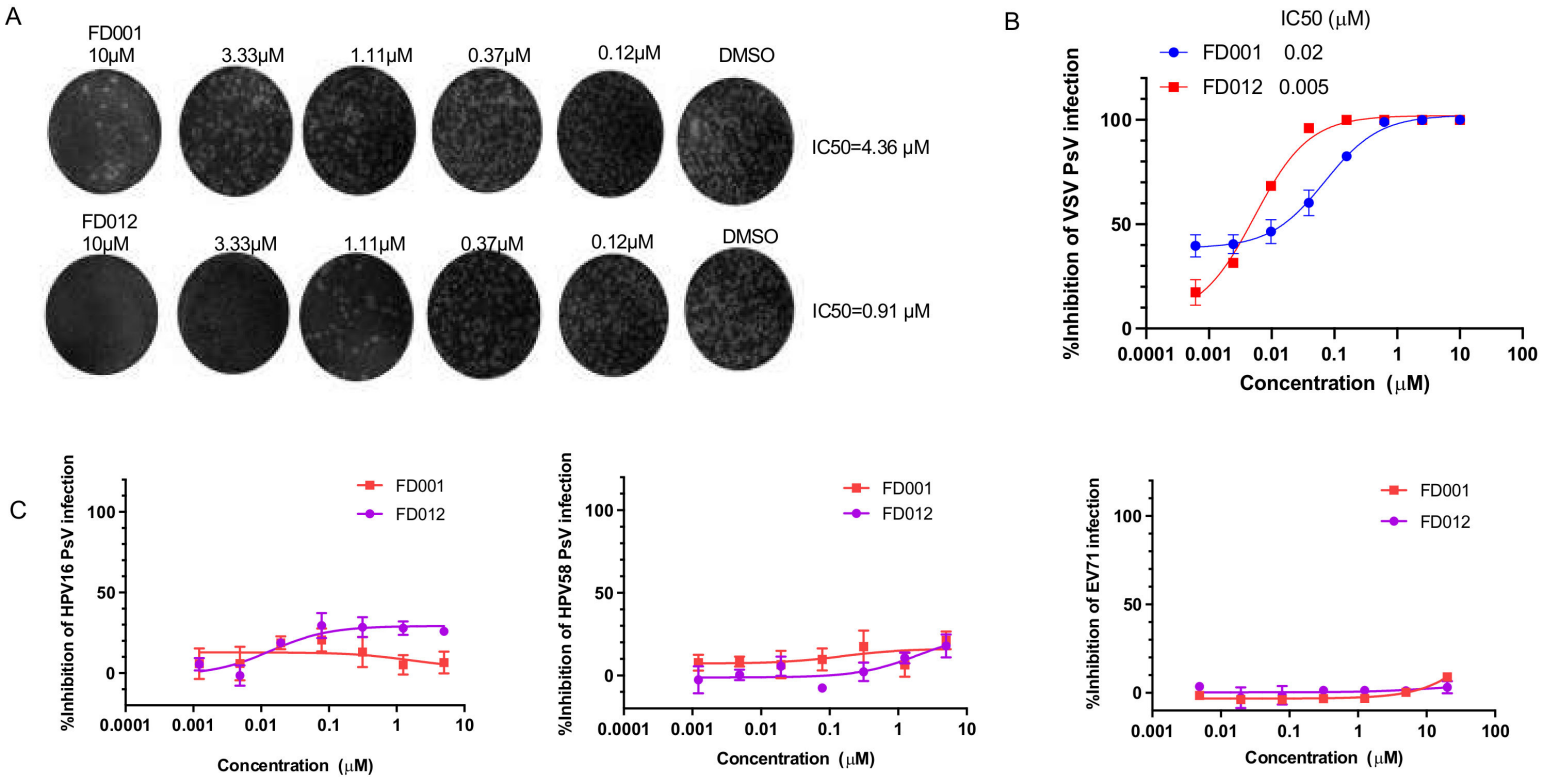
FD-compounds inhibited infection of enveloped viruses with class II and III viral fusion protein, but not nonenveloped viruses. (**A**) FD001 and FD012 inhibited authentic ZIKV PRVABC59 (2015/Puerto Rico) infection in Vero E6 cells; (**B**) FD001 and FD012 inhibited VSV PsV infection in Huh-7 cells; (**C**) FD001 and FD012 could not inhibit infection of the nonenveloped HPV PsV in Hela cells and authentic EV71 in RD cells. Data are means ± SD of triplicate samples from a representative experiment.

In addition, we also evaluated the activity of FD-compounds against infection of class II enveloped viruses, such as ZIKV, and class III enveloped viruses, such as VSV. We found that FD001 and FD012 could effectively inhibit infection of authentic ZIKV and pseudotyped VSV (Figure 3A,B). Interestingly, we found that they were not effective against infection of the nonenveloped viruses, such as the authentic EV71 and pseudotyped HPV (Figure 3C). On the basis of the above results, we conclude that these compounds have antiviral effects against infection of class I, II, and III enveloped viruses, but not nonenveloped viruses.

### 3.5. FD-Compounds Exerted Antiviral Effects through Multiple Mechanisms of Action

**Figure 4:**
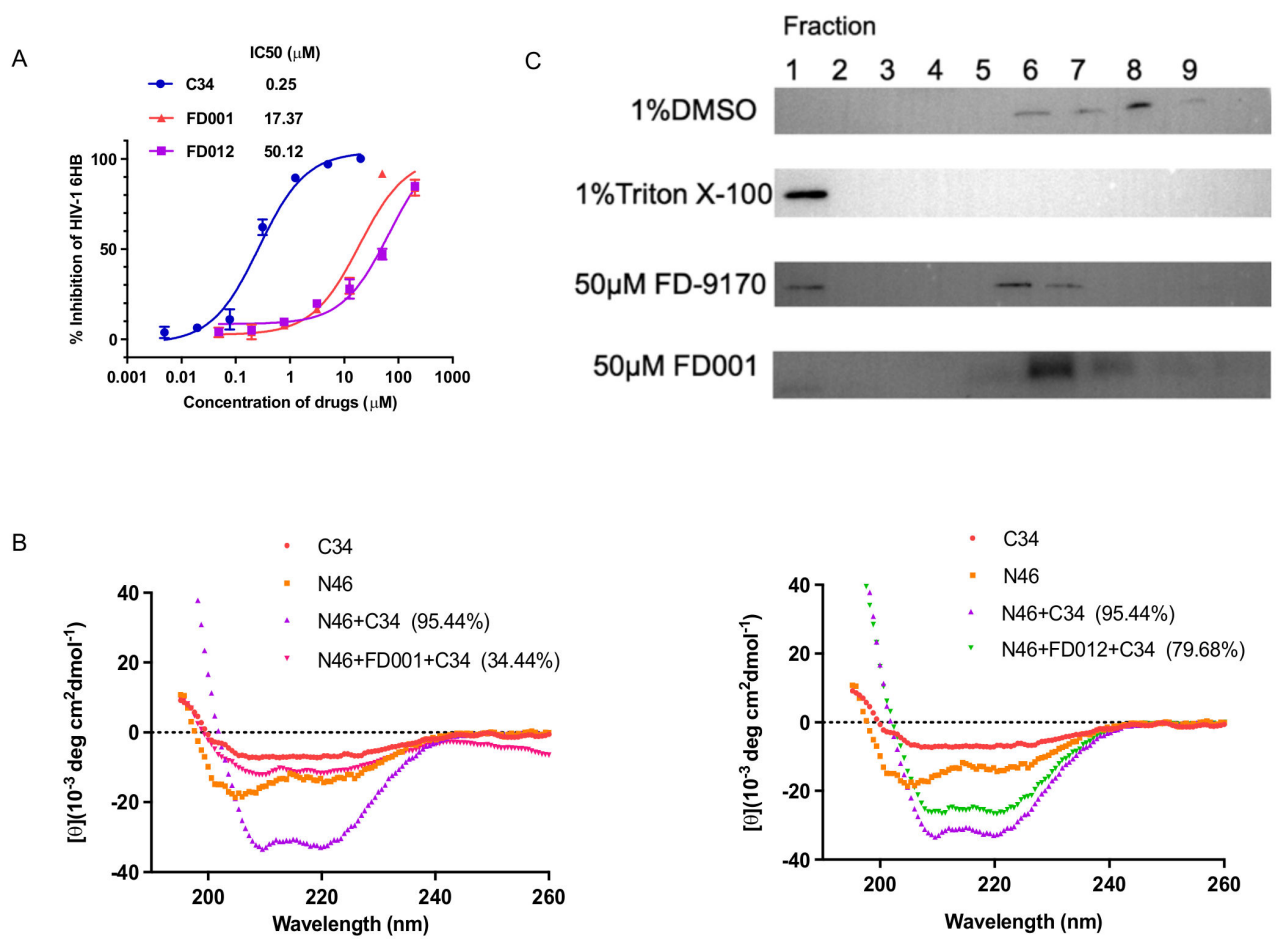
FD-compounds inhibited HIV-1 6-HB formation and did not disrupt the integrity of the viral membrane. FD-compounds inhibited HIV-1 6-HB formation between N46 and C34 peptides measured by ELISA (**A**) and CD spectroscopy (**B**); compound FD001 did not disrupt the integrity of the viral membrane (**C**).

**Figure 5:**
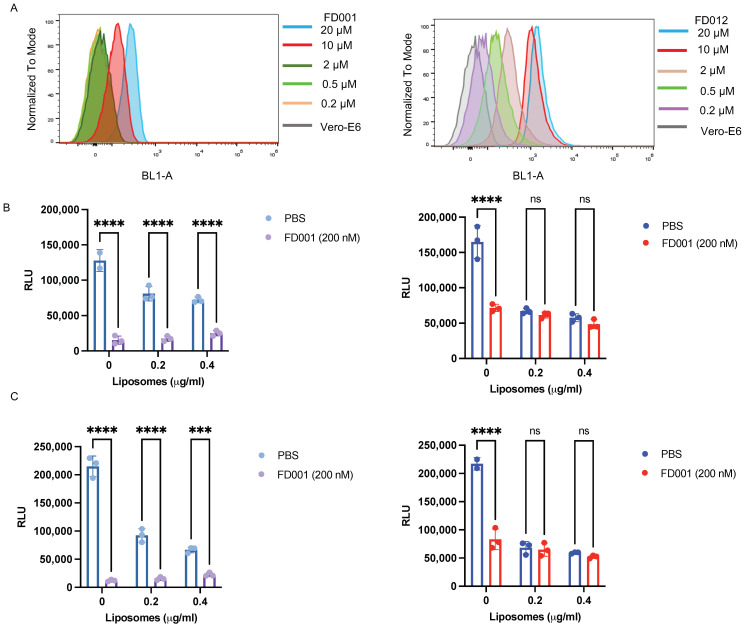
FD-compounds may target the lipid membrane of the enveloped virus. (**A**) FD-compounds bind to Vero E6 cells in a concentration-dependent manner; (**B**) HIV-1 Bal.01 PsVs were pretreated with FD001, and then added into target cells in the presence of recombinant unilamellar liposomes (size = 400 nm, 8:2 molar ratio of phosphatidylcholine (PC) to cholesterol) (**left**), while FD001 was pretreated with unilamellar liposomes (400 nm) and virus successively, and then added into target cells (**right**); (**C**) the liposome size was 100 nm, and the other conditions were the same as in B. Two-way ANOVA was used in the statistical analysis. **** and *** mean *p* < 0.0001 and *p* < 0.001, respectively.

Given that FD-compounds are effective against enveloped viruses, we wondered whether they acted on a common target of enveloped viruses. As shown in Figure 4A, FD001 and FD012 inhibited HIV-1 6-HB between N46 and C34 peptides in a concentration-dependent manner. Similar results were obtained by circular dichroism (CD) spectroscopy. The addition of FD001 and FD012 reduced the α-helical content of the complex formed by N46 and C34 peptides from 95.44% to 34.44% and 79.68%, respectively (Figure 4B). However, as the 6-HB sequence of different enveloped viruses varies, we turned our attention to the viral membrane because some reported broad-spectrum antiviral inhibitors act by destroying membrane structure [32]. To test this idea, HIV-1 PsV was pretreated with FD001 compound and then subjected to sucrose density gradient centrifugation. The result shows that the positive control FD-9170 peptide had a band distribution similar to that of the 1% Triton X-100 group [26], while the FD001-treated group had a distribution similar to the negative group (1% DMSO) (Figure 4C). This result confirms that compound treatment did not destroy the integrity of the lipid membrane. It should be noted that one reported compound, LJ001, has a similar structure to FD001 [33].LJ001 binds to lipid membranes and exerts antiviral activity by reducing the fluidity of lipid membranes by generating singlet oxygen [34,35]. As shown in Figure 5A, after treating Vero E6 cells with FD001 or FD012 compound, the fluorescence intensity of the cells was positively correlated with compound concentration since the compound has green fluorescence. Furthermore, when recombinant unilamellar liposomes were added to HIV-1 PsV pretreated with FD001, the compound showed good antiviral activity compared with the PBS group. However, when FD001 was pretreated with liposomes, its inhibitory activity against HIV-1 PsV was no different from that of the PBS group (Figure 5B,C). These results suggest pretreatment with liposomes invalidated the activity of FD001, which may act on the viral lipid membrane to exert antiviral activity. Therefore, we speculate that the mechanism of action of FD001 is similar to that of LJ001.

## 4. Discussion

It is difficult to develop mature antiviral treatments or vaccines in a short period of time for most viral infectious diseases. Designing specific antiviral drugs is time-consuming and expensive. Therefore, evaluating the potential effects of marketed drugs or potential inhibitors or screening natural product libraries is a relatively rapid and economical method [36,37]. There are several FDA-approved antiviral drugs, such as famciclovir for the treatment of herpes simplex virus infection [38], ganciclovir for the treatment of cytomegalovirus infection [39,40], and various reverse transcriptase inhibitors for the treatment of HIV infection [41,42], based on natural products [36]. Moreover, some natural products show promise in inhibiting SARS-CoV-2 infection [43,44,45], indicating that they are a good choice for the development of antiviral drugs. Due to the frequent outbreaks of viral infectious diseases, the development of broad-spectrum antiviral drugs is valuable and can also be used to prevent possible future viral outbreaks. Most viral infectious diseases are caused by enveloped viruses, which have bilayer lipid membranes derived from host cells, and viral envelope proteins are distributed on the surface of these lipid membranes. However, the sequence of the viral fusion protein differs among the enveloped viruses. Therefore, an effective target for the development of broad-spectrum anti-enveloped virus inhibitors may be the viral lipid membrane.

Previously, we demonstrated that FD001 could inhibit HIV-1 infection of target cells [16]. In this study, we designed a series of FD001 analogs and found that they had inhibitory and inactivating activities against highly pathogenic HCoVs, such as SARS-CoV, MERS-CoV, and SARS-CoV-2 (Figure 1 and Table 2), and pathogenic HCoVs, e.g., HCoV-OC43 (Supplementary Figure S1). Importantly, these compounds could effectively inhibit infection of the highly pathogenic HCoVs, including SARS-CoV-2 VOCs, such as Alpha (B.1.1.7), Beta (B.1.351), Gamma (P.1), Delta (B.1.617.2), and Omicron (B.1.1.529) (Figure 1). FD-compounds were also effective against other highly pathogenic viruses with class I viral fusion protein, such as influenza viruses H1N1 and H3N2 (Supplementary Figure S2), H5N1 and H7N9 (Supplementary Table S2), EBOV, NiV, and LASV (Supplementary Table S3). These compounds could also effectively inhibit infection of viruses with class II viral fusion protein, such as ZIKV (Figure 3A), or class III viral fusion protein, such as VSV (Figure 3B). On the other hand, they could not inhibit the infection of nonenveloped viruses, such as HPV and EV71 (Figure 3C).

Compound FD001 binds to cells in a concentration-dependent manner (Figure 5A), but it does not exert antiviral activity by targeting the proteins in the host cells, as removal of the unbound compounds by washing the compound-treated cells resulted in no inhibition of the viral infection (Figure 2A). However, pretreatment of the enveloped virus with FD-compounds made it noninfective (Figure 2B), indicating that these compounds act on the viral particles, not the host cells. Further results indicate that the FD001 treatment cannot destroy the structural integrity of the viral lipid membrane (Figure 4C). That is, even though virions were pretreated with FD001, the compound still showed effective antiviral activity in the presence of recombinant unilamellar liposomes (Figure 5B, left). However, when FD001 was pretreated with recombinant unilamellar liposomes, its antiviral activity was blocked (Figure 5B, right). These results indicate that the FD-compounds may target the lipid membrane of the enveloped virus to exert their broad-spectrum antiviral activity. Compounds could effectively inhibit the formation of 6-HB between HIV-1 N46 and C34 peptide (Figure 4A,B), suggesting that, like furanyl methylidene rhodamine, these analogous compounds could inhibit HIV-1 fusion with and entry into the host cell by targeting the HIV-1 gp41 NHR domain.

In summary, we reported a series of small-molecule compounds that are highly effective against enveloped viruses, including SARS-CoV-2 and its variants, through multiple mechanisms of action. These compounds are expected to be further developed to meet the challenge of existing or possible future enveloped virus infections.

## Figures and Tables

**Table 1:** Structure of FD001 and its analogs.

Compounds	R	MW(Da)
FD001	H	470.0
FD007	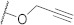	524.01
FD008	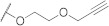	568.06
FD009	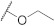	514.01
FD010	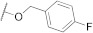	594.07
FD012	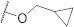	540.05
FD013	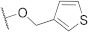	582.11

**Table 2:** Antiviral activity of FD001 and its analogs against SARS-CoV and MERS-CoV PsV infection.

Compound	CC_50_ (µM)	IC_50_ (µM)	SI	EC_50_ (µM)	SI
			CC_50_/IC_50_		CC_50_/EC_50_
SARS-CoV PsV infection in hACE2-293T cells					
FD001	28.84 ± 1.24	0.18 ± 0.02	160.22	1.58 ± 0.21	18.25
FD007	33.11 ± 1.02	0.11 ± 0.01	301.00	1.54 ± 0.14	21.50
FD008	23.44 ± 0.78	0.05 ± 0.01	468.80	0.19 ± 0.02	123.37
FD009	17.37 ± 0.64	0.03 ± 0.01	579.00	0.12 ± 0.01	144.75
FD010	20.42 ± 1.00	0.29 ± 0.03	70.41	0.19 ± 0.03	107.47
FD012	12.30 ± 0.34	0.02 ± 0.01	615.00	0.12 ± 0.01	102.50
FD013	18.62 ± 0.23	0.05 ± 0.01	372.40	0.10 ± 0.01	186.20
MERS-CoV PsV infection in Huh-7 cells					
FD001	42.65 ± 1.31	0.29 ± 0.03	147.07	1.12 ± 0.16	38.08
FD007	>50	0.20 ± 0.03	>250	0.89 ± 0.09	>56.18
FD008	38.90 ± 1.22	0.31 ± 0.02	125.48	0.69 ± 0.13	56.38
FD009	27.54 ± 1.43	0.28 ± 0.02	98.36	0.29 ± 0.02	94.97
FD010	44.67 ± 1.26	0.12 ± 0.01	372.25	0.04 ± 0.01	1116.75
FD012	18.62 ± 0.35	0.11 ± 0.01	169.27	0.05 ± 0.01	372.40
FD013	30.90 ± 0.63	0.05 ± 0.01	618.00	0.03 ± 0.01	1030.00

The IC_50_ of mAb 33G4 was 0.03 μg/mL for inhibiting SARS-CoV PsV infection in hACE2-293T cells, while the IC_50_ of mAb m336 was 0.006 μg/mL for inhibiting MERS-CoV PsV infection in Huh-7 cells.

## Data Availability

Not applicable.

## Supplementary Materials

The following supporting information can be downloaded at: www.mdpi.com/article/10.3390/v14030489/s1, Figure S1: FD001 and FD012 inhibited authentic HCoV-OC43 infection; Figure S2: FD001 inhibited authentic IAV infection; Table S1: Information on the infection of different target cells by different viruses; Table S2: Inhibitory activity of compounds against H5N1 and H7N9 PsV; Table S3: Inhibitory activity of compound FD001 and FD012 against class I enveloped viruses.
