# Supplementary material for: The Analogs of Furanyl Methylidene Rhodanine Exhibit Broad-Spectrum Inhibitory and Inactivating Activities against Enveloped Viruses, including SARS-CoV-2 and Its Variants
Source: Viruses. 2022 Feb 27;14(3):489. doi: 10.3390/v14030489 (PMC8954792; doi:10.3390/v14030489)
Supplement: Supplementary file 1 [file viruses-14-00489-s001.zip › viruses-1599536-supplementary.pdf]

Supplementary Materials

The Analogs of Furanyl Methylidene Rhodanine Exhibit Broad-spectrum Inhibitory and Inactivating Activities Against Enveloped Viruses, Including SARS-CoV-2 and Its Variants

Jing Pu[1#], Xiaoyang He[2#], Wei Xu[1], Cong Wang[1], Qiaoshuai Lan[1], Chen Hua[1], Kai Wang[1], Lu Lu[1,*] and Shibo Jiang [1,*]

[1] Key Laboratory of Medical Molecular Virology (MOE/NHC/CAMS), School of Basic Medical Sciences; Shanghai Institute of Infectious Disease and Biosecurity; Biosafety Level 3 Laboratory, Shanghai Medical College, Shanghai Frontiers Science Center of Pathogenic Microbes and Infection, Fudan University, Shanghai, China; 17111010015@fudan.edu.cn (J.P.); xuwei11@fudan.edu.cn (W.X.); congwang1111@163.com (C.W.); 18111010010@fudan.edu.cn (Q.L.); 16211010047@fudan.edu.cn (C.H.); 16111010090@fudan.edu.cn (K.W.);

[2] Beijing Institute of Radiation Medicine, Beijing, China; hexiaoyang@aliyun.com (X.H.)

[#] These authors contributed equally to this work.

* Correspondence: shibojiang@fudan.edu.cn (S.J.); lul@fudan.edu.cn (L.L.); Tel.: +86-21-5423-7673 (S.J.); +86-21-5423-7671 (L.L.)

Supplementary Table S1. Information on the infection of different target cells by different viruses

Virus	Virus titer	Target Cell	Culture condition
	PsV		
SARS-CoV-2	100 TCID50	hACE2-293T	48 h
SARS-CoV-2 variants	100 TCID50	Caco2	48 h
SARS-CoV	100 TCID50	hACE2-293T	48 h
MERS-CoV	100 TCID50	Huh-7	48 h
VSV	100 TCID50	Huh-7	48 h
EBOV	100 TCID50	Huh-7	48 h
LASV	100 TCID50	Huh-7	48 h
H5N1	100 TCID50	MDCK	48 h
H7N9	100 TCID50	MDCK	48 h
HPV	100 TCID50	HeLa	48 h

NiV	100 TCID50	U87	48 h
	Authentic virus		
SARS-CoV-2 WT (nCoV-SH01)	0.01 MOI	Vero-E6	72 h
SARS-CoV-2 delta variant	0.0025 MOI	Vero-E6	72 h
H3N2 (A/Victoria/361/2011)	0.001 MOI	MDCK	72 h
H1N1 (A/California/04/2009)	0.001 MOI	MDCK	72 h
ZIKV PRVABC59 (2015/Puerto Rico)	0.001 MOI	Vero-E6	120 h
EV71 (VR-1432)	100 TCID50	RD	24 h
HCoV-OC43 (VR-1558)	100 TCID50	RD	72 h

Supplementary Table S2. Inhibitory activity of compounds against H5N1 and H7N9 PsV.

Compound	CC50 (μM)	IC50 (μM)	
		H5N1	H7N9
FD001	>50	0.26±0.03	0.85±0.21
FD007	>50	0.47±0.11	2.13±0.20
FD008	>50	0.51±0.14	2.51±0.33
FD009	>50	0.15±0.04	1.41±0.27
FD010	>50	0.97±0.21	5.88±0.61
FD012	41.69	0.15±0.01	0.23±0.02
FD013	>50	0.26±0.02	3.54±0.89

Supplementary Table S3. Inhibitory activity of compound FD001 and FD012 against class I enveloped viruses.

Virus	IC50 (μM)

	FD001	FD012
EBOV (Zaire)	1.04±0.11	0.09±0.02
EBOV (Sudan)	0.39±0.04	0.19±0.02
NiV	0.41±0.07	0.07±0.01
LASV	0.44±0.10	0.32±0.04

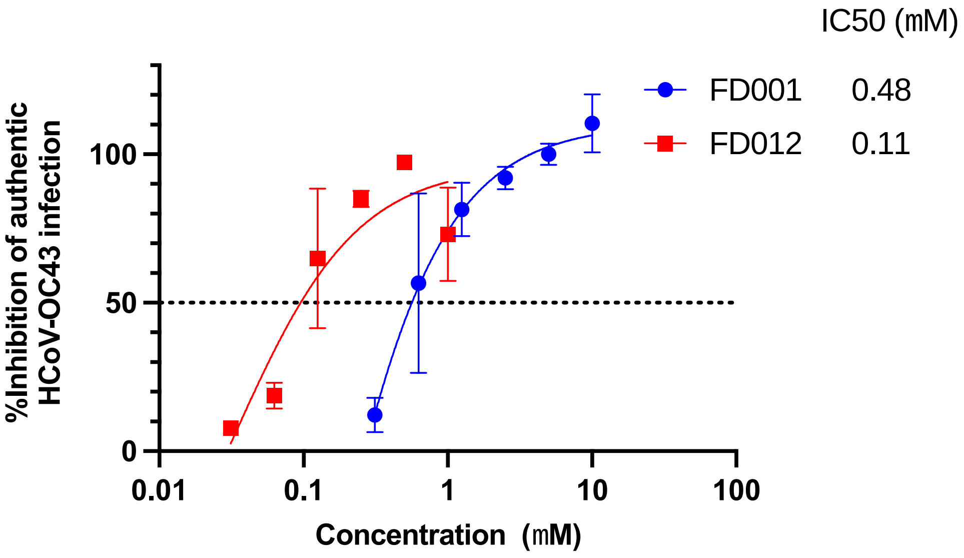

Supplementary Figure S1. FD001 and FD012 inhibited authentic HCoV-OC43 infection. The inhibitory activity of FD001 and FD012 against authentic HCoV-OC43 infection was assessed with CCK-8 assay.

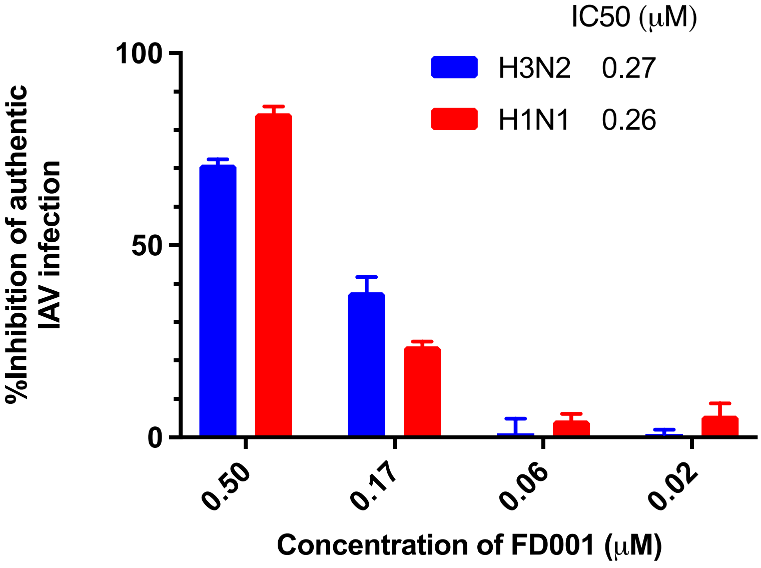

Supplementary Figure S2. FD001 inhibited authentic IAV infection. The inhibitory activity of FD001 against authentic IAV H3N2 and H1N1 infection in MDCK cells was evaluated with plaque reduction assay.

Chemical synthesis of furanyl methylidene rhodamine analogs

All chemical reagents were commercially available and used without any further purification unless other mentioned. The reaction process was monitored by analytical TLC (silica gel GF254). Flash column chromatography was carried out with Teledyne Isco Combiflash Rf200 purification. ^{1}H-NMR (400 MHz), ^{13}C-NMR (100 MHz) were recorded on a JEOL JNM-ECA-400 spectrometer with tetramethylsilane as internal standard. Mass spectra of molecules were measured on an Agilent 1260-G6230A mass spectrometer. Purity of the compounds was determined by HPLC, HPLC analyses were performed on a Waters 2695 with Waters 2487 detector using the following: Kromsil C18 analytical column (4.6 mm × 250 mm, particle size 5 μm) with dectection at 210 nm; solvent, CH_3CN/H_2O containing 0.01%TFA (v/v) (gradual elution: 70-95%) at a flow rate of 1.5 mL/min at 30 °C.

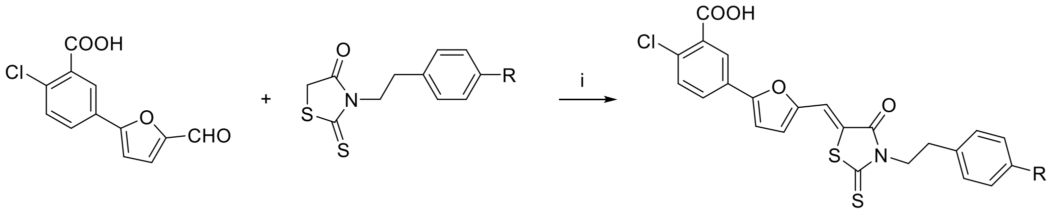

1 **2a–f** **FD007–010 and FD012–013**

Scheme S1. The synthesis procedure for **FD001** analogs **FD007–010 and FD012–013**. Reagents and conditions: (i) $CH_3CO_2NH_4$, EtOH, reflux, 2 h.

The general procedure for preparation of FD007–010 and **FD012–013.**

To a suspension of **1** (1 equiv.) and **2a–f** (1.1 equiv.) in 10 mL EtOH was added catalytic amount of $CH_3CO_2NH_4$ (10% mol), the reaction mixture was stirred for 2 h under reflux. After cooling to room temperature, the precipitate was filtered out to afford the target compounds **FD007–010** and **FD012–013**.

(Z)-2-chloro-5-(5-((4-oxo-3-(4-(prop-2-yn-1-yloxy)phenethyl)-2-thioxothiazolidin-5-ylidene)methyl)furan-2-yl)benzoic acid (FD007)

Starting **1** (100 mg, 0.4 mmol) and **2a** (116 mg, 0.4 mmol) to afford 170 mg of **FD007** as red solid, 81% yield, HPLC purity 99.21%. ^{1}H NMR (400MHz, DMSO-d_6) δ 8.11 (s, 1H), 7.88 (d, J = 8.0 Hz, 1H), 7.70 (d, J = 8.8 Hz, 1H), 7.66 (s, 1H), 7.47 (d, J = 3.6 Hz, 1H), 7.39 (d, J = 3.6 Hz, 1H), 7.17 (d, J = 8.4 Hz, 2H), 6.93 (d, J = 8.4 Hz, 2H), 4.76 (s, 2H), 4.22 (t, J = 8.0 Hz, 2H), 3.56 (s, 1H), 2.91 (t, J

= 8.0 Hz, 2H); ^{13}C NMR (100MHz, DMSO-d_6) δ 193.57, 166.80, 166.31, 156.21, 155.96, 149.63, 131.42, 130.29, 129.69, 127.32, 126.66, 125.80, 123.08, 118.99, 118.16, 114.90, 111.33, 79.33, 78.17, 55.33, 45.44, 31.32; ESI-MS (*m/z*) 522.03 [M - H]$^-$.

(Z)-2-chloro-5-(5-((4-oxo-3-(4-(2-(prop-2-yn-1-yloxy)ethoxy)phenethyl)-2-thioxothiazolidin-5-ylidene)methyl)furan-2-yl)benzoic acid (FD008)

Starting **1** (90 mg, 0.36 mmol) and **2b** (120 mg, 0.36 mmol) to afford 140 mg of **FD008** as red solid, 68% yield, HPLC purity 98.03%. ^1H NMR (400MHz, DMSO-d6) δ 8.09 (d, J = 2.4 Hz, 1H), 7.84 (dd, J = 8.4, 2.0 Hz, 1H), 7.68 (d, J = 8.4 Hz, 1H), 7.63 (s, 1H), 7.43 (d, J = 4.0 Hz, 1H), 7.36 (d, J = 4.0 Hz, 1H), 7.13 (d, J = 8.4 Hz, 2H), 6.88 (d, J = 8.4 Hz, 2H), 4.21-4.16 (m, 4H), 4.07-4.05 (m, 2H), 3.76-3.74 (m, 2H), 3.49-3.47 (t, J = 3.0 Hz, 1H), 2.90 (t, J = 7.2 Hz, 2H); 13C NMR (100MHz, DMSO-d6) δ 193.59, 166.47, 166.32, 157.15, 155.98, 149.71, 131.72, 131.69, 129.74, 127.65, 126.12, 123.03, 119.11, 118.15, 114.48, 111.49, 80.22, 77.41, 66.72, 66.70, 57.61, 44.50, 31.30; ESI-HRMS (m/z) 568.0652 [M+H]+ , 585.0911 [M + Na]+.

(Z)-2-chloro-5-(5-((3-(4-ethoxyphenethyl)-4-oxo-2-thioxothiazolidin-5-ylidene)methyl)furan-2-yl)benzoic acid (FD009)

Starting **1** (100 mg, 0.4 mmol) and **2c** (100 mg, 0.36 mmol) to afford 84 mg of **FD009** as red solid, 45% yield, HPLC purity 98.26%. ^1H NMR (400MHz, DMSO-d_6) δ 7.92 (d, *J* = 2.0 Hz, 1H), 7.74 (dd, *J* = 8.4, 2.0 Hz, 1H), 7.63 (s, 1H), 7.57 (d, *J* = 8.4 Hz, 1H), 7.38-7.35 (m, 2H), 7.12 (d, *J* = 8.7 Hz, 2H), 6.85 (d, *J* = 8.7 Hz, 2H), 5.76 (s, 1H), 4.20 (t, *J* = 7.8 Hz, 2H), 3.99 (q, *J* = 13.7, 6.7 Hz, 2H), 2.89 (t, *J* = 7.8 Hz, 2H), 1.31 (t, *J* = 7.2 Hz, 3H); ^{13}C NMR (100MHz, DMSO-d_6) δ 193.58, 166.32, 157.25, 156.88, 149.44, 130.74, 129.69, 129.31, 126.98, 124.95, 123.20, 118.62, 118.24, 114.40, 110.88, 62.86, 45.49, 31.29, 14.66; ESI-MS (*m/z*) 514.05 [M + H]$^+$.

(Z)-2-chloro-5-(5-((3-(4-((4-fluorobenzyl)oxy)phenethyl)-4-oxo-2-thioxothiazolidin-5-ylidene)methyl)furan-2-yl)benzoic acid (FD010)

Starting **1** (85 mg, 0.30 mmol) and **2d** (100 mg, 0.28 mmol) to afford 62 mg of **FD010** as red solid, 37% yield, HPLC purity 99.36%. ^1H NMR (400MHz, DMSO-d_6) δ 8.12 (d, *J* = 2 Hz, 1H), 7.88 (dd, *J* = 8.4, 2.0 Hz, 1H), 7.70 (d, *J* = 8.7 Hz, 2H), 7.63 (s, 1H), 7.45-7.36 (m, 3H), 7.27-7.24 (m, 2H), 7.15-7.13 (m, 3H), 6.95 (d, *J* = 8.7 Hz, 2H), 5.08 (s, 2H), 4.21 (t, *J* = 7.6 Hz, 2H), 2.91 (t, *J* = 7.6 Hz, 2H);

^{13}C NMR (100MHz, DMSO-d_6) δ 193.14, 166.28, 165.88, 162.97, 160.54, 156.42, 155.73, 149.22, 120.72, 139.65, 131.05, 130.04, 129.97, 129.49, 139.33, 126.93, 126.39, 125.46, 123.04, 122.62, 118.60, 117.71, 114.44, 113.96, 113.84, 113.62, 110.94, 67.82, 45.02, 30.86; ESI-MS (*m/z*) 594.05 $[M + H]^+$.

(Z)-2-chloro-5-(5-((3-(4-(cyclopropylmethoxy)phenethyl)-4-oxo-2-thioxothiazolidin-5-ylidene)methyl)furan-2-yl)benzoic acid (FD012)

Starting **1** (88 mg, 0.35 mmol) and **2e** (100 mg, 0.33 mmol) to afford 62 mg of **FD012** as red solid, 35% yield, HPLC purity 98.28%. ^1H NMR (400 MHz, DMSO-d_6) δ 13.77 (br s, 1H), 8.20 (d, *J* = 2.2 Hz, 1H), 7.94 (dd, *J* = 8.4, 2.2 Hz, 1H), 7.75 (d, *J* = 8.4 Hz, 1H), 7.64 (s, 1H), 7.48 (d, *J* = 3.6 Hz, 1H), 7.37 (d, *J* = 3.6 Hz, 1H), 7.11 (d, *J* = 8.8 Hz, 2H), 6.84 (d, *J* = 8.8 Hz, 2H), 4.19 (t, *J* = 7.6 Hz, 2H), 3.76 (d, *J* = 7.0 Hz, 2H), 2.89 (t, *J* = 7.8 Hz, 2H), 1.20-1.16 (m, 1H), 0.56-0.52 (m, 2H), 0.30-0.28 (m, 2H); ^{13}C NMR (100MHz, DMSO-d_6) δ 195.56, 166.30, 157.37, 155.87, 149.71, 131.77, 129.66, 129.27, 127.49, 126.23, 122.96, 119.15, 118.09, 114.48, 111.53, 71.89, 45.49, 31.27; ESI-MS (*m/z*) 540.07 $[M + H]^+$.

(Z)-2-chloro-5-(5-((4-oxo-3-(4-(thiophen-3-ylmethoxy)phenethyl)-2-thioxothiazolidin-5-ylidene)methyl)furan-2-yl)benzoic acid (FD013)

Starting **1** (79 mg, 0.32 mmol) and **2f** (100 mg, 0.29 mmol) to afford 37 mg of **FD013** as red solid, 22% yield, HPLC purity 99.30%. ^1H NMR (400 MHz, DMSO-d_6) δ 7.97 (s, 1H), 7.75 (d, *J* = 8.0 Hz, 1H), 7.65 (s, 1H), 7.60 (d, *J* = 8.4 Hz, 1H), 7.55-7.52 (m, 2H), 7.41-7.37 (m, 2H), 7.17-7.12 (m, 3H), 6.94 (d, *J* = 8.8 Hz, 2H); ^{13}C NMR (100MHz, DMSO-d_6) δ 193.93, 166.69, 157.32, 149.73, 138.33, 130.99, 130.06, 127.95, 127.27, 126.94, 125.05, 124.27, 123.57, 118.91, 118.60, 115.12, 111.18, 65.05, 45.79, 31.60; ESI-MS (*m/z*) 626.04 $[M + H]^+$, 579.26 $[M - CO_2]^+$.

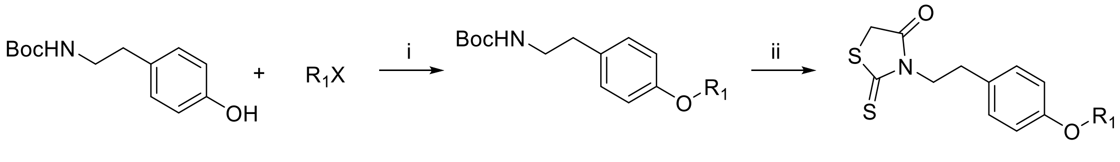

3 **4a–f** **5a–f** **2a–f**

X = Br or OMs

Scheme S2. The synthesis procedure of Rhodanine derivatives **2a–f**. Reagents and conditions: (i) K_2CO_3, DMF, 80 °C, 12 h; (ii) (1) AcCl, MeOH, r.t., 24 h; (2) bis(carboxymethyl) trithiocarbonate, Et_3N, *i*-PrOH, MW, 90 °C, 45 min.

The general procedure for preparation of Rhodanine derivatives 5a–f

To a suspension of *N*-Boc-tyramine **3** (1 equiv.) and K_2CO_3 (1.1-3 equiv.) in 5 mL/mmol DMF was added corresponding **4a–f** (0.88-3 equiv.). The reaction mixture was stirred at 80 °C for 12 h. The reaction mixture was cooled to room temperature and extracted with EtOAc, the organic phases were collected and washed with brine, dried over Na_2SO_4 to finally be filtered and concentrated, and the resulting residue was purified by flash column chromatography (0-40% EtOAc/petroleum ether) to afford Rhodanine derivatives **5a–f**.

***Tert*-butyl 4-(prop-2-yn-1-yloxy)phenethylcarbamate (5a)**

Starting with **3** (3.5 g, 14.7 mmol) and Propargyl bromide **4a** (80% w/w in toluene, 2.0 mL, 17 mmol) to afford 2.65 g of **5a** as pale-yellow oil, 66% yield. ^1H NMR (400 MHz, $CDCl_3$) δ 7.14 (d, J = 8.4 Hz, 2H), 6.93 (d, J = 8.4 Hz, 2H), 4.68 (d, J = 2.8 Hz, 2H), 4.53 (br s, 1H), 3.55 (q, J = 2.4 Hz, 2H), 2.74 (t, J = 6.4 Hz, 2H), 2.53 (t, J = 2.4 Hz, 1H), 1.43 (s, 9H).

***Tert*-butyl 4-(2-(prop-2-yn-1-yloxy)ethoxy)phenethylcarbamate (5b)**

Starting with **3** (1.46 g, 6.15 mmol) and 2-(prop-2-yn-1-yloxy)ethyl methanesulfonate **4b** (1.64 g, 9.23 mmol) to afford 0.8 g of **5b** as white oil, 41% yield. ^1H NMR (400 MHz, $CDCl_3$) δ 7.11 (d, J = 8.8 Hz, 2H), 6.87 (d, J = 8.4 Hz, 2H), 4.51 (br s, 1H), 4.28 (d, J = 2.0 Hz, 2H), 4.15-4.13 (m, 2H), 3.91-3.89 (m, 2H), 3.96 (q, J = 6.0 Hz, 2H), 1.43 (s, 9H).

***Tert*-butyl 4-ethoxyphenethylcarbamate (5c)**

Starting with **3** (600 mg, 2.5 mmol) and bromoethane **4c** (0.58 mL，7.5 mmol) to afford 550 mg of **5c** as white solid, 82% yield. ^1H NMR (400 MHz, $CDCl_3$) δ 7.10 (d, J = 8.4 Hz, 2H), 6.85 (d, J = 8.4 Hz, 2H), 4.51 (br s, 1H), 4.01 (q, J = 6.8 Hz, 2H), 3.35-3.31 (m, 2H), 2.74 (t, J = 7.2 Hz, 2H), 1.42-1.38 (m, 12H).

***Tert*-butyl 4-((4-fluorobenzyl)oxy)phenethylcarbamate (5d)**

Starting with **3** (500 mg, 2.1 mmol) and 1-(bromomethyl)-4-fluorobenzene **4d** (0.55 mL，6.3 mmol) to afford 550 mg of **5d** as white solid, 93% yield. ^1H NMR (400 MHz, $CDCl_3$) δ 7.37-7.32 (m, 1H), 7.20-7.10 (m, 4H), 7.03-6.99 (m, 1H), 6.92 (d, J = 8.8 Hz, 2H), 5.04 (s, 2H), 4.53 (br s, 1H), 3.37-3.34 (m, 2H), 2.75 (d, J = 6.8 Hz, 2H), 1.43 (s, 9H).

***Tert*-butyl 4-(cyclopropylmethoxy)phenethylcarbamate (5e)**

Starting with **3** (800 mg，3.37 mmol) and (bromomethyl)cyclopropane **4e** (0.4 mL, 4.05 mmol) to afford 876 mg of **5e** as white solid, 81% yield. ^{1}H NMR (400 MHz, $CDCl_3$) δ 7.10 (d, J = 8.4 Hz, 2H), 6.86-6.83 (m, 2H), 4.51 (s, 1H), 3.79 (d, J = 7.0 Hz, 2H), 3.36-3.33 (m, 2H), 2.74 (t, J = 6.7 Hz, 2H), 1.43 (s, 9H), 1.29-1.25 (m, 1H), 0.66-0.62 (m, 2H), 0.36-0.32 (m, 2H).

***Tert*-butyl (4-(thiophen-3-ylmethoxy)phenethyl)carbamate (5f)**

Starting with **3** (606 mg, 2.6 mmol) and 3-(bromomethyl)thiophene **4f** (400 mg, 2.3 mmol) to afford 700 mg of **5f** as white solid, 82% yield. ^{1}H NMR (400MHz, $CDCl_3$) δ 7.35-7.31 (m, 2H), 7.15 (dd, J = 1.2, 5.0 Hz, 1H), 7.11 (d, J = 8.4 Hz, 2H), 6.91 (d, J = 8.4 Hz, 2H), 5.04 (s, 2H), 4.52 (br s, 1H), 3.34 (m, 2H), 2.74 (t, J = 6.7 Hz, 2H), 1.43 (s, 9H).

3-(4-(Prop-2-yn-1-yloxy)phenethyl)-2-thioxothiazolidin-4-one (2a)

To a suspension of **5a** (2.65 g, 9.6 mmol) in 6 mL MeOH was slowly added AcCl (2.1 mL, 28.8 mmol) at 0 ^{o}C, the reaction mixture was allowed to warm to room temperature for 24 h. The reaction mixture was concentrated to remove the solvents and added 15 mL ether, the residue was precipitated and filtered out to give amine hydrochloride intermediate (1.83 g, 90% yield). Then, the amine hydrochloride intermediate (100 mg, 0.47 mmol) was added into a suspension of bis(carboxymethyl) trisulfide carbonate (114 mg, 0.5 mmol) and Et_3N (70 μL, 0.5 mmol) in 5 mL *i*-PrOH. The reaction mixture was heated in a microwave to 90 ^{o}C for 45 min. After the reaction reached completion, the mixture was extracted using EtOAc, the organic phases were collected, washed with brine, and dried over Na_2SO_4 to finally be filtered and concentrated, the resulting residue was purified by flash column chromatography (0-40% EtOAc/petroleum ether) to afford 80 mg of rhodanine derivative **2a** as pale-yellow solid, 58% yield. ^{1}H NMR (400MHz, $CDCl_3$) δ 7.21 (d, J = 8.4 Hz, 2H), 6.93 (d, J = 8.4 Hz, 2H), 4.68 (d, J = 2.8 Hz, 2H), 4.18-4.14 (m, 2H), 3.94 (s, 2H), 2.90-2.86 (m, 2H), 2.53 (t, J = 2.4 Hz, 1H); 2.89 (t, J = 8.0 Hz, 2H).

3-(4-(2-(Prop-2-yn-1-yloxy)ethoxy)phenethyl)-2-thioxothiazolidin-4-one (2b)

2b was synthesized by the analogous procedure for preparation of **2a**, starting with **5b** (800 mg, 2.5 mmol) to afford amine hydrochloride intermediate (610 mg, 95% yield). Treatment with the intermediate (500 mg, 2.0 mmol) gave 370 mg of **2b** as pale-yellow solid, 56% yield. ^{1}H NMR

(400MHz, $CDCl_3$) δ 7.17 (d, *J* = 8.8 Hz, 2H), 6.87 (d, *J* = 8.8 Hz, 2H), 4.28 (d, *J* = 2.0 Hz, 2H), 4.18-4.12 (m, 2H), 3.93-3.89 (m, 2H), 2.75 (t, *J* = 7.2 Hz, 2H), 2.47 (t, *J* = 6.4 Hz, 1H).

DY-4-21 3-(4-Ethoxyphenethyl)-2-thioxothiazolidin-4-one (2c)

2c was synthesized by the analogous procedure for preparation of **2a**, starting with **5c** (550 mg, 2.07 mmol) to afford amine hydrochloride intermediate (440 mg, 95% yield). Treatment with the intermediate (300 mg, 1.5 mmol) gave 180 mg of **2c** as pale-yellow solid, 43% yield. ^{1}H NMR (400 MHz, $CDCl_3$) δ 7.17 (d, *J* = 8.8 Hz, 2H), 6.84 (d, *J* = 8.8 Hz, 2H), 4.18-4.14 (m, 2 H), 4.04 (q, *J* = 7.2 Hz, 2H), 3.93 (s, 2H), 2.89-2.85 (m, 2H), 1.42 (t, *J* = 7.2 Hz, 3H).

3-(4-((4-Fluorobenzyl)oxy)phenethyl)-2-thioxothiazolidin-4-one (2d)

2d was synthesized by the analogous procedure for preparation of **2a**, starting with **5d** (700 mg, 2.03 mmol) to afford amine hydrochloride intermediate (565 mg, 99% yield). Treatment with the intermediate (400 mg, 1.4 mmol) gave 180 mg of **2d** as pale-yellow solid, 35% yield. ^{1}H NMR (400 MHz, $CDCl_3$) δ 7.37-7.32 (m, 1H), 7.20-7.14 (m, 4H), 7.03-6.99 (m, 1H), 6.92-6.88 (m, 2H), 5.04 (s, 2H), 4.18-4.14 (m, 2H), 3.92 (s, 2H), 3.90-2.86 (m, 2H).

3-(4-(Cyclopropylmethoxy)phenethyl)-2-thioxothiazolidin-4-one (2e)

2e was synthesized by the analogous procedure for preparation of **2a**, starting with **5e** (1.0 g, 3.43 mmol) to afford amine hydrochloride intermediate (780 mg, 100% yield). Treatment with the intermediate (500 mg, 2.21 mmol) gave 297 mg of **2e** as pale-yellow solid, 44% yield. ^{1}H NMR (400 MHz, $CDCl_3$) δ 7.17-7.14 (m, 2H), 6.86-6.82 (m, 2H), 4.17-4.13 (m, 2H), 3.93 (s, 2H), 3.78 (d, *J* = 6.8 Hz, 2H), 2.89-2.85 (m, 2H), 1.28-1.24 (m, 1H), 0.66-0.62 (m, 2H), 0.36-0.32(m, 2H).

3-(4-(Thiophen-3-ylmethoxy)phenethyl)-2-thioxothiazolidin-4-one (2f)

2f was synthesized by the analogous procedure for preparation of **2a**, starting with **5f** (500 mg, 1.5 mmol) to afford amine hydrochloride intermediate (363 mg, 94% yield). Treatment with the intermediate (260 mg, 1.10 mmol) gave 242 mg of **2f** as pale-yellow solid, 63% yield.^{1}H NMR (400 MHz, $CDCl_3$) δ 7.35-7.32 (m, 2H), 7.18-7.14 (m, 3H), 6.92 (d, *J* = 8.4 Hz, 2H), 5.05 (s, 2H), 4.18-4.14 (m, 2H), 3.92 (s, 2H), 2.90 (m, 2H).
